# Supplementary material for: Negative-Pressure Wound Therapy for Prevention of Sternal Wound Infection after Adult Cardiac Surgery: Systematic Review and Meta-Analysis
Source: J Clin Med. 2022 Jul 22;11(15):4268. doi: 10.3390/jcm11154268 (PMC9330054; doi:10.3390/jcm11154268)
Supplement: Supplementary file 1 [file jcm-11-04268-s001.zip › jcm-1813585-supplementary.pdf]

Supplementary Materials

Negative-Pressure Wound Therapy for Prevention of Sternal Wound Infection after Adult Cardiac Surgery: Systematic Review and Meta-Analysis

Fausto Biancari [1,2,*], Grazia Santoro [1], Federica Provenzano [1], Leonardo Savarese [1], Francesco Iorio [1], Salvatore Giordano [1], Carlo Zebele [1] and Giuseppe Speziale [3]

Content:

Supplementary Table S1. 2020 PRISMA checklist.

Supplementary Table S2. Main characteristics of studies included in quantitative analysis.

Supplementary Table S3. Summary of quality assessment of studies included in quantitative analysis.

Supplementary Figure S1. Literature search flowchart.

Supplementary Figure S2. Funnel plot of studies reporting on any sternal wound infection.

Supplementary Figure S3. Funnel plot of studies reporting on superficial sternal wound infection.

Supplementary Figure S4. Funnel plot of studies reporting on deep sternal wound infection.

Supplementary Figure S5. Forest plots of leave-one-out meta-analysis.

Supplementary Table S1. 2020 PRISMA checklist.

Section and Topic	Item #	Checklist item	Location where item is reported (page)
**TITLE**			
Title	1	Identify the report as a systematic review.	1
**ABSTRACT**			
Abstract	2	See the PRISMA 2020 for Abstracts checklist.	OK
**INTRODUCTION**			
Rationale	3	Describe the rationale for the review in the context of existing knowledge.	3
Objectives	4	Provide an explicit statement of the objective(s) or question(s) the review addresses.	3
**METHODS**			
Eligibility criteria	5	Specify the inclusion and exclusion criteria for the review and how studies were grouped for the syntheses.	4
Information sources	6	Specify all databases, registers, websites, organisations, reference lists and other sources searched or consulted to identify studies. Specify the date when each source was last searched or consulted.	3
Search strategy	7	Present the full search strategies for all databases, registers and websites, including any filters and limits used.	3
Selection process	8	Specify the methods used to decide whether a study met the inclusion criteria of the review, including how many reviewers screened each record and each report retrieved, whether they worked independently, and if applicable, details of automation tools used in the process.	4
Data collection process	9	Specify the methods used to collect data from reports, including how many reviewers collected data from each report, whether they worked independently, any processes for obtaining or confirming data from study investigators, and if applicable, details of automation tools used in the process.	4
Data items	10a	List and define all outcomes for which data were sought. Specify whether all results that were compatible with each outcome domain in each study were sought (e.g. for all measures, time points, analyses), and if not, the methods used to decide which results to collect.	5
	10b	List and define all other variables for which data were sought (e.g. participant and intervention characteristics, funding sources). Describe any assumptions made about any missing or unclear information.	5
Study risk of bias assessment	11	Specify the methods used to assess risk of bias in the included studies, including details of the tool(s) used, how many reviewers assessed each study and whether they worked independently, and if applicable, details of automation tools used in the process.	5
Effect measures	12	Specify for each outcome the effect measure(s) (e.g. risk ratio, mean difference) used in the synthesis or presentation of results.	5
Synthesis methods	13a	Describe the processes used to decide which studies were eligible for each synthesis (e.g. tabulating the study intervention characteristics and comparing against the planned groups for each synthesis (item #5)).	4-5
	13b	Describe any methods required to prepare the data for presentation or synthesis, such as handling of missing summary statistics, or data conversions.	4-5
	13c	Describe any methods used to tabulate or visually display results of individual studies and syntheses.	5
	13d	Describe any methods used to synthesize results and provide a rationale for the choice(s). If meta-analysis was performed, describe the model(s), method(s) to identify the presence and extent of statistical heterogeneity, and software package(s) used.	4-5
	13e	Describe any methods used to explore possible causes of heterogeneity among study results (e.g. subgroup analysis, meta-regression).	4-5
	13f	Describe any sensitivity analyses conducted to assess robustness of the synthesized results.	5
Reporting bias assessment	14	Describe any methods used to assess risk of bias due to missing results in a synthesis (arising from reporting biases).	5
Certainty assessment	15	Describe any methods used to assess certainty (or confidence) in the body of evidence for an outcome.	5
**RESULTS**			
Study selection	16a	Describe the results of the search and selection process, from the number of records identified in the search to the number of studies included in the review, ideally using a flow diagram.	5-6

Section and Topic	Item #	Checklist item	Location where item is reported (page)
	16b	Cite studies that might appear to meet the inclusion criteria, but which were excluded, and explain why they were excluded.	Suppl. Fig. 1
Study characteristics	17	Cite each included study and present its characteristics.	5, suppl. tables
Risk of bias in studies	18	Present assessments of risk of bias for each included study.	Suppl. Tab. 3
Results of individual studies	19	For all outcomes, present, for each study: (a) summary statistics for each group (where appropriate) and (b) an effect estimate and its precision (e.g. confidence/credible interval), ideally using structured tables or plots.	Text, tables, figures
Results of syntheses	20a	For each synthesis, briefly summarise the characteristics and risk of bias among contributing studies.	Text, tables, figures
	20b	Present results of all statistical syntheses conducted. If meta-analysis was done, present for each the summary estimate and its precision (e.g. confidence/credible interval) and measures of statistical heterogeneity. If comparing groups, describe the direction of the effect.	Text, tables, figures
	20c	Present results of all investigations of possible causes of heterogeneity among study results.	Text, tables, figures
	20d	Present results of all sensitivity analyses conducted to assess the robustness of the synthesized results.	5-6
Reporting biases	21	Present assessments of risk of bias due to missing results (arising from reporting biases) for each synthesis assessed.	-
Certainty of evidence	22	Present assessments of certainty (or confidence) in the body of evidence for each outcome assessed.	5-6
**DISCUSSION**			
Discussion	23a	Provide a general interpretation of the results in the context of other evidence.	6-8
	23b	Discuss any limitations of the evidence included in the review.	6-8
	23c	Discuss any limitations of the review processes used.	6-8
	23d	Discuss implications of the results for practice, policy, and future research.	6-8
**OTHER INFORMATION**			
Registration and protocol	24a	Provide registration information for the review, including register name and registration number, or state that the review was not registered.	3
	24b	Indicate where the review protocol can be accessed, or state that a protocol was not prepared.	3
	24c	Describe and explain any amendments to information provided at registration or in the protocol.	-
Support	25	Describe sources of financial or non-financial support for the review, and the role of the funders or sponsors in the review.	8
Competing interests	26	Declare any competing interests of review authors.	8
Availability of data, code and other materials	27	Report which of the following are publicly available and where they can be found: template data collection forms; data extracted from included studies; data used for all analyses; analytic code; any other materials used in the review.	-

Supplementary Table S2. Main characteristics of studies included in quantitative analysis.

Author	Year of publication	Country	Randomized study	Propensity score matching	Randomized, allocated or PS matching study	Inclusion criteria	NPWT dressing	ST dressing	Planned time of NPWT (days)	Definition of SWI
Brega	2021	Italy	No	No	No	Two of the following criteria: diabetes on medical treatment, BMI>30 kg/m2, COPD, diabetes, eGFR < 60 ml/min, BIMA grafting	Avelle (ConvaTec, Deeside, UK)	Hydrocolloid and carboxymethyl cellulose, or traditional gauze dressing	7	Center for Disease Control and Prevention
Grauhan	2013	Germany	No	No	Yes	BMI>30 kg/m2, absence of preoperative signs of inflammation	Prevena (3M Corporate, St. Paul, MN, USA)	NR	6-7	Center for Disease Control and Prevention
Grauhan	2014	Germany	No	No	No	NR	Prevena (3M Corporate, St. Paul, MN, USA)	NR	6-7	NR
Myllykangas	2021	Finland	No	Yes	Yes	Any CABG, BMI>30, kg/m2, diabetes	PICO (Smith+Nephew Inc., Andover, MA, USA)	Not reported	6-7	Center for Disease Control and Prevention
Rashed	2021	Hungary	Yes	No	Yes	BMI>30 kg/m2, diabetes, PVD, COPD	VivanoTec (Hartmann Ltd., Budapest, Hungary)	Not reported	5	Horan TC et al. Am J Infect Control. 2008; 36:309332
Ruggieri	2019	France	No	Yes	Yes	Isolated CABG, BIMA grafting	Prevena (3M Corporate, St. Paul, MN, USA)	Tegaderm (3M Corporate, St. Paul, MN, USA)	5-7	Center for Disease Control and Prevention
Santarpino	2015	Germany	No	No	No	Isolated CABG, BIMA grafting	Prevena (3M Corporate, St. Paul, MN, USA)	Cosmopor E (Paul Hartmann AG, Heidenheim, Germany)	5	NR
Suelo-Calanao	2020	United Kingdom	No	No	No	NR	Prevena (3M Corporate, St. Paul, MN, USA)	Opsite Post-op (Smith+Nephew Inc., Andover, MA, USA)	5	Jones G, et al. Ann Surg 1997;225:766-776.
Tabley	2020	France	No	No	No	NR	PICO (Smith+Nephew Inc., Andover, MA, USA)	NR	NR	NR
Witt-Majchrzak	2014	Poland	Yes	No	Yes	CABG	Prevena (3M Corporate, St. Paul, MN, USA)	NR	6	European Centre for Disease Prevention and Control

BIMA=bilateral internal mammary artery; CABG=coronary artery bypass grafting; NPWT=negative pressure wound therapy; NR=not reported; PS=propensity score; ST=standard treatment.

Supplementary Table S3. Summary of quality assessment of studies included in quantitative analysis.

Author	Year of publication	1. Was the research question or objective in this paper clearly stated and appropriate?	2. Was the study population clearly specified and defined?	3. Did the authors include a sample size justification?	4. Were controls selected or recruited from the same or similar population that gave rise to the cases (including the same timeframe)?	5. Were the definitions, inclusion and exclusion criteria, algorithms or processes used to identify or select cases and controls valid, reliable, and implemented consistently across all study participants?	6. Were the cases clearly defined and differentiated from controls?	7. If less than 100 percent of eligible cases and/or controls were selected for the study, were the cases and/or controls randomly selected from those eligible?	8. Was there use of concurrent controls?	9. Were the investigators able to confirm that the exposure/risk occurred prior to the development of the condition or event that defined a participant as a case?	10. Were the measures of exposure/risk clearly defined, valid, reliable, and implemented consistently (including the same time period) across all study participants?	11. Were the assessors of exposure/risk blinded to the case or control status of participants?	12. Were key potential confounding variables measured and adjusted statistically in the analyses? If matching was used, did the investigators account for matching during study analysis?	Quality rating
Brega	2021	Yes	Yes	No	No	No	No	No	NR	Yes	No	No	No	Poor
Grauhan	2013	Yes	Yes	No	Yes	Yes	Yes	No	NR	Yes	Yes	NR	No	Fair
Myllykangas	2021	Yes	Yes	No	No	Yes	Yes	No	NR	Yes	No	No	Yes	Poor
Rashed	2021	Yes	Yes	No	Yes	Yes	Yes	Yes	NR	Yes	No	NR	No	Fair
Ruggieri	2019	Yes	Yes	No	Yes	Yes	Yes	No	NR	Yes	No	No	Yes	Poor
Suelo-Calanao	2020	Yes	Yes	No	No	No	No	No	NR	Yes	No	No	No	Poor
Witt-Majchrzak	2014	Yes	Yes	No	Unclear	No	Yes	Unclear	NR	Yes	Yes	No	No	Fair
Santarpino	2015	Yes	Yes	No	No	No	No	No	NR	Yes	No	No	No	Poor
Tabley	2020	Yes	Yes	No	No	No	No	No	NR	Yes	No	No	No	Poor

NR=not reported.

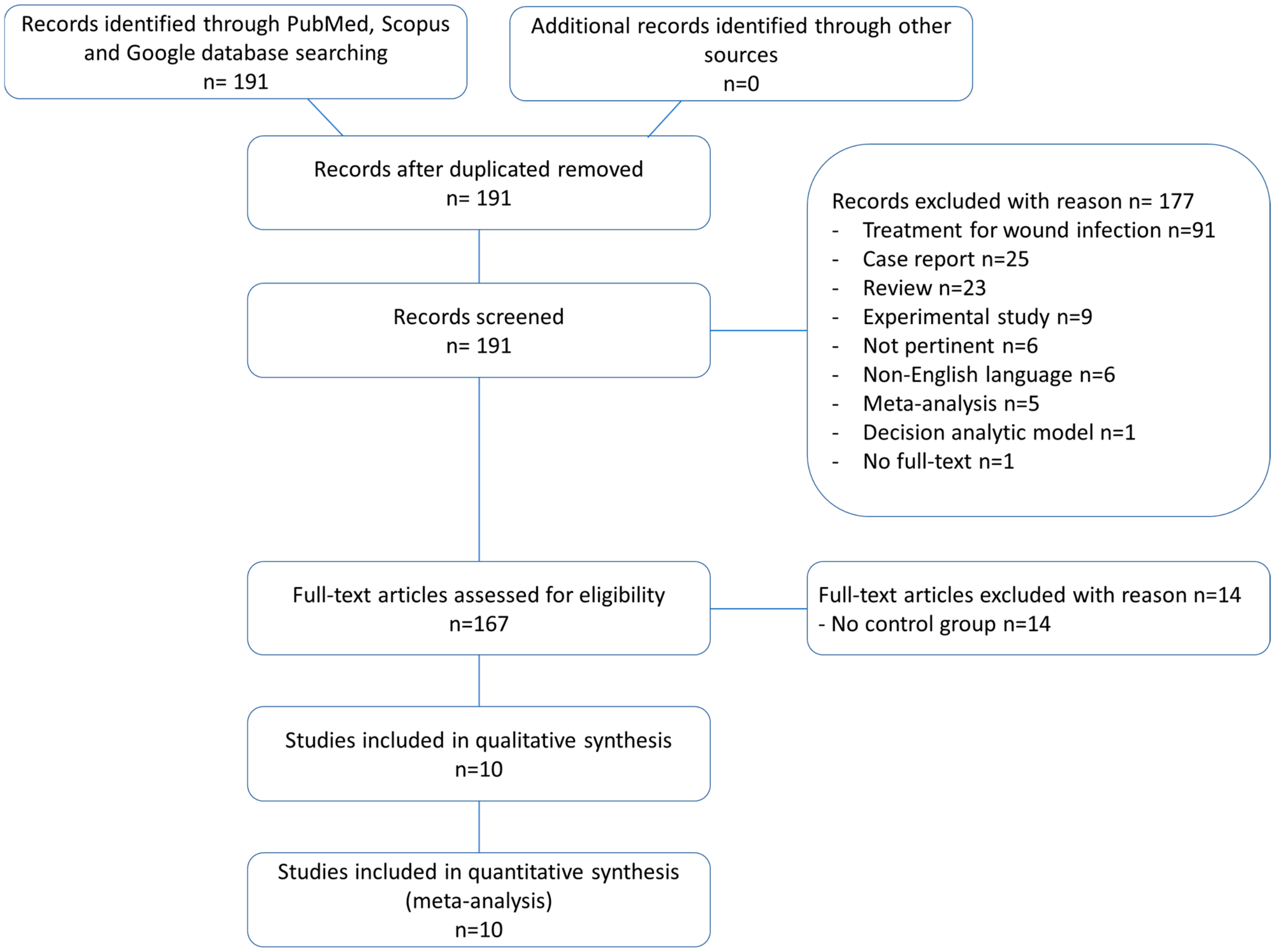

Supplementary Figure S1. Literature search flowchart.

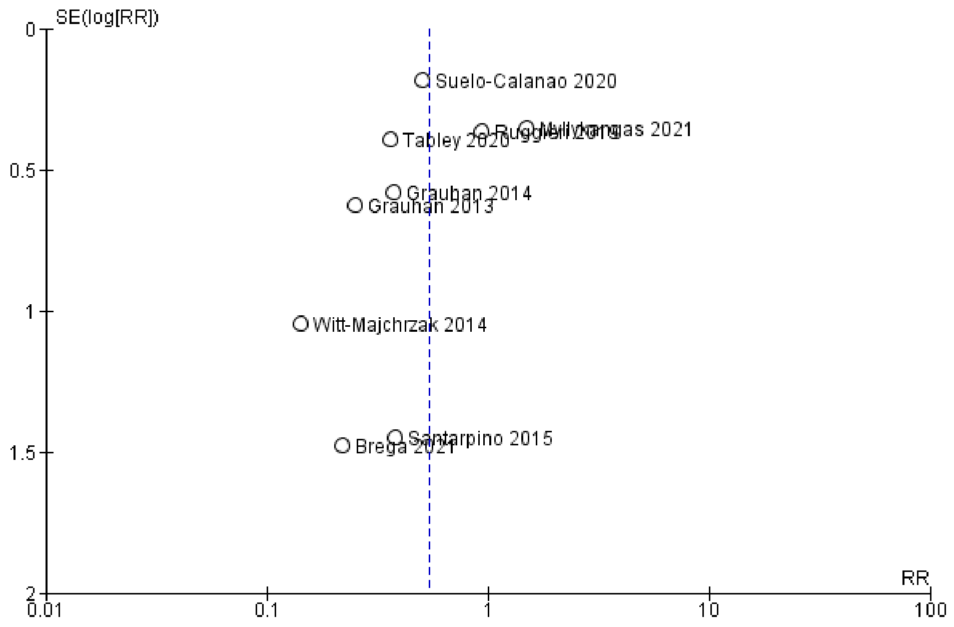

Supplementary Figure S2. Funnel plot of studies reporting on any sternal wound infection.

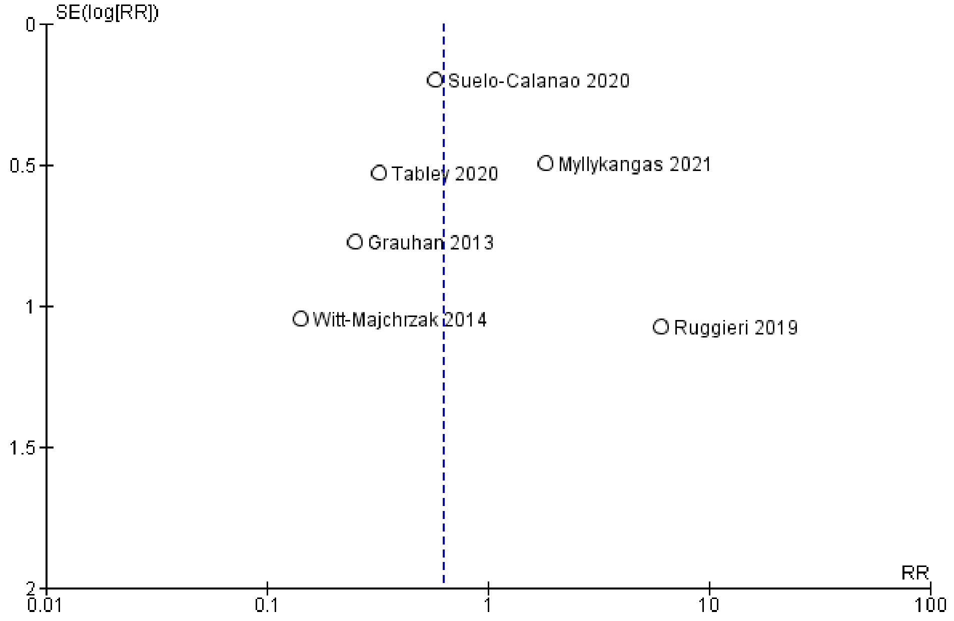

Supplementary Figure S3. Funnel plot of studies reporting on superficial sternal wound infection.

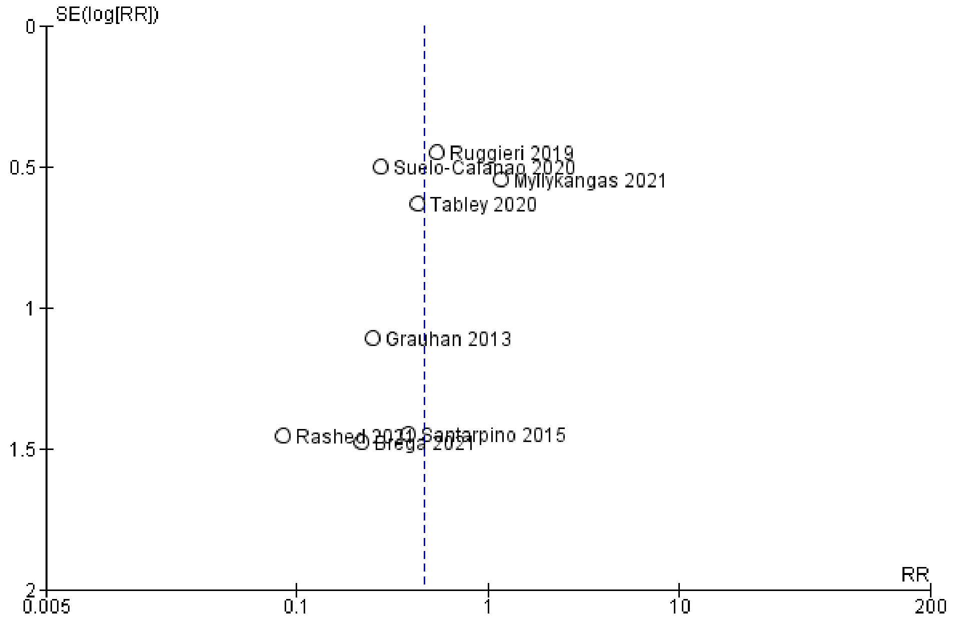

Supplementary Figure S4. Funnel plot of studies reporting on deep sternal wound infection.

Any sternal wound infection

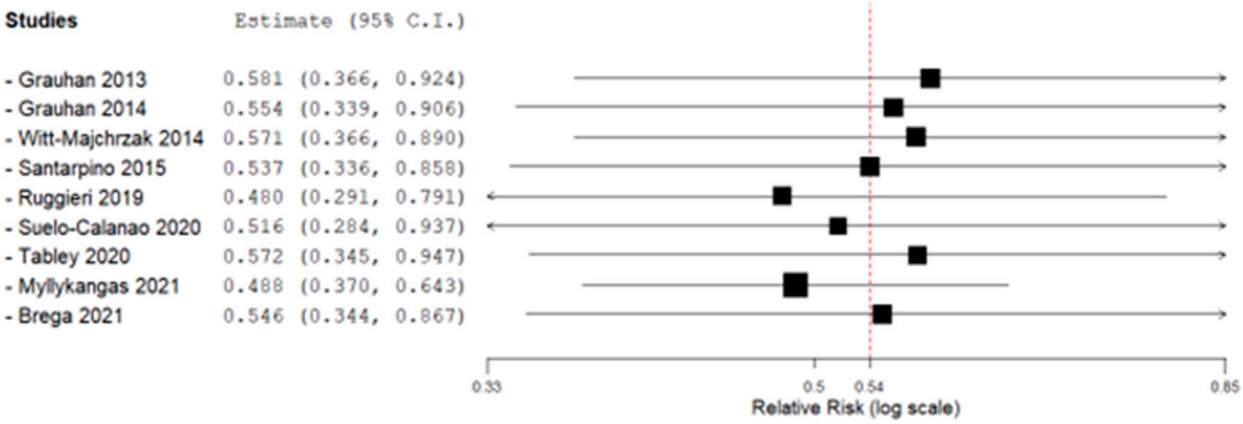

Superficial sternal wound infection

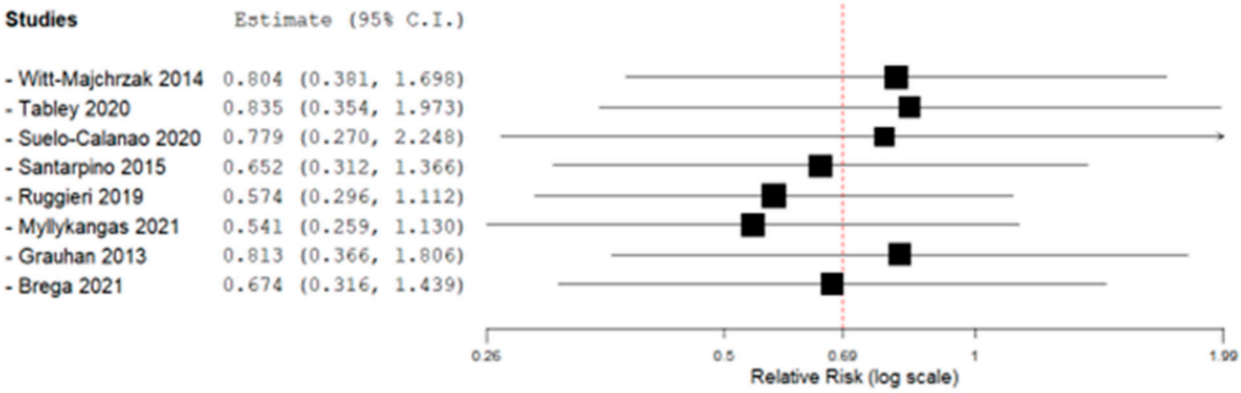

Deep sternal wound infection

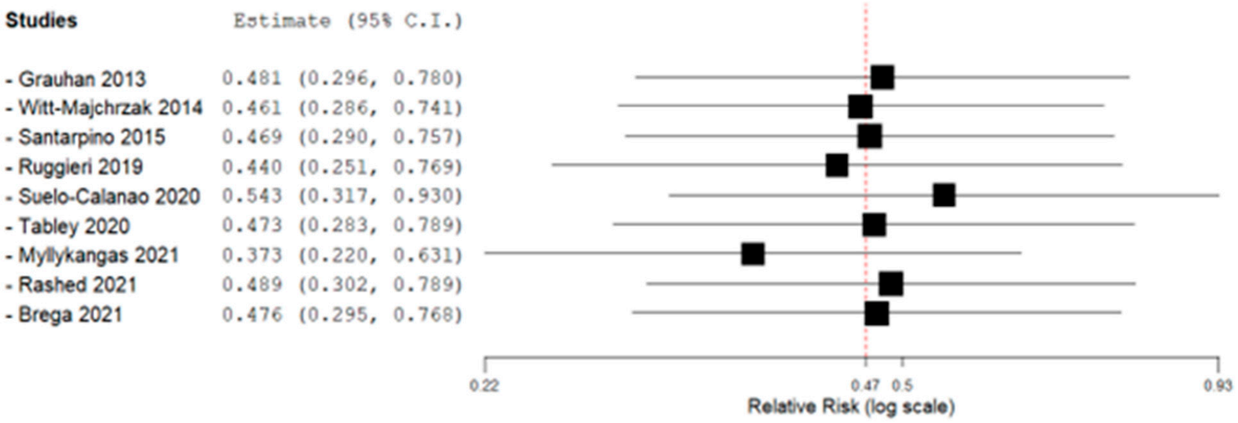

Supplementary Figure S5. Forest plots of leave-one-out meta-analysis.
